# Effect of combining a health program with a microfinance-based self-help group on health behaviors and outcomes

**DOI:** 10.1016/j.puhe.2015.07.010

**Published:** 2015-11

**Authors:** S. Saha, M. Kermode, P.L. Annear

**Affiliations:** a Nossal Institute for Global Health, University of Melbourne, Australia; b Indian Institute of Public Health Gandhinagar, Gujarat, India

**Keywords:** Microfinance, Self-help group, Difference-in-difference, Health behaviours, India

## Abstract

**Objectives:**

Women's participation in microfinance-based self-help groups (SHGs) and the resultant social capital may provide a basis to address the gap in health attainment for poor women and their children. We investigated the effect of combining a health program designed to improve health behaviours and outcomes with a microfinance-based SHG program.

**Design:**

A mixed method study was conducted among 34 villages selected from three blocks or district subdivisions of India; one in Gujarat, two in Karnataka.

**Methods:**

A set of 17 villages representing new health program areas were pair-matched with 17 comparison villages. Two rounds of surveys were conducted with a total of 472 respondents, followed by 17 key informant interviews and 17 focus group discussions.

**Results:**

Compared to a matched comparison group, women in SHGs that received the health program had higher odds of delivering their babies in an institution (OR: 5.08, 95% CI 1.21–21.35), feeding colostrum to their newborn (OR: 2.83, 95% CI 1.02–5.57), and having a toilet at home (OR: 1.53, 95% CI 0.76–3.09). However, while the change was in the expected direction, there was no statistically significant reduction in diarrhoea among children in the intervention community (OR: 0.86, 95% CI 0.42–1.76), and the hypothesis that the health program would result in decreased out-pocket expenditures on treatment was not supported.

**Conclusion:**

Our study found evidence that health programs implemented with microfinance-based SHGs is associated with improved health behaviours. With broad population coverage of SHGs and the social capital produced by their activities, microfinance-based SHGs may provide an avenue for addressing the health needs of poor women.

## Highlights

• Combining a health program with SHGs is associated with an improvement in key health behaviours in the study population.
• However, the program led to no significant improvement in diarrhoea among children, and no effect on spending on treatment.
• The structure of SHGs and duration of association played a role in promoting trust between members and the host organization.
• Working with SHGs provides a basis for addressing the gap in health attainment for women and their children.

## Introduction

Self-help groups (SHGs), usually comprising 10–20 individuals (predominantly women) and organized to save money and obtain microfinance, are an important initiative that provide access to capital and promote livelihoods among the rural poor in India. These SHGs are promoted extensively through government and non-government organizations and were estimated to reach 93 million members in 2012.^1^ The SHG structure facilitates significant face-to-face interaction between members and promotes mutual trust, solidarity and social capital.^2^, ^3^ Women's participation in microfinance-based SHGs and the resultant social capital may provide a basis for improving health outcomes and addressing the gap in health attainment for women and their children.

In a previous study in India, we found that the presence of an SHG in a village was associated with improved maternal and child health knowledge and practice.^4^ Elsewhere, a clustered randomized trial among indigenous communities in Jharkhand and Odisha states of India found that newborn babies born in communities with an SHG had a significantly improved likelihood of surviving the first six weeks of life compared to babies born to analogous households in non-SHG communities.^5^, ^6^ Within a broader holistic community development initiative in the early 1970s in Jamkhed, Maharashtra state of India, a program was implemented among women's groups in which one woman from each group was trained as a health worker and funds were provided to assist the group members in the event of health emergencies. During the first 20 years, the project showed a reduction in infant mortality rate from 176 to 19 per 1000 live births, and the birth rate declined from 40 to 20 per 1000 people. Access to antenatal care, safe delivery and immunization was nearly universal and malnutrition declined from 40% to less than 5% in the study population.^7^, ^8^ A study of women's participation in savings groups in Bangladesh found that membership of microfinance programs was associated with an increased probability of children being fully immunized.^9^ A study of the microcredit forum of BRAC, a non-government development organization in Bangladesh, found a significant positive effect of membership in the forum on maternal knowledge of prenatal care, increase use of contraceptive use, and a decline in fertility.^10^, ^11^

However, despite this evidence, using these mechanisms to address the health needs of the poor does not appear to be a high priority for health planners in India. And while India has large programs – both government and non-government organized – to promote microfinance schemes to poor women, there is limited evidence on the role of health programs attached to microfinance-based SHGs in improving health outcomes of the poor. This paper reports on the findings from a field study designed to investigate whether combining a health program with a microfinance-based SHG program improves health behaviours and outcomes.

## Methods

### Study design and sites

To assess the effect of combining a health program with a microfinance-based SHG program, a difference-in-difference analysis was conducted through two rounds of surveys to collect baseline and one-year follow-up data from intervention and matched comparison group. The quantitative field study was conducted during 2012 and 2013, followed by a qualitative investigation of the contextual factors and challenges associated with the health program. The study was conducted among 34 villages selected from three blocks or district subdivisions of India: Dahegam in Gujarat, Udupi and Gadag in Karnataka.

Women in these villages had access to microfinance programs from two organizations: the Self Employed Women's Association (SEWA) in Gujarat, and the Shri Kshetra Dharamstala Rural Development Project (SKDRDP) in Karnataka. Both organizations provided a health program for member groups. In the case of SEWA, the health programs were organized as member-owned cooperatives, and included primary health care delivered through stationery and mobile health camps, health education and training, and the production and marketing of traditional medicines. The SEWA health program was supported by funding from philanthropic organizations. SEWA also offered insurance schemes that included health cover for its members. The health package covered hospitalisation costs up to Rs. 2000 (US $33) annually for an individual, with options for family coverage up to Rs. 25,000 (US $416) per year, against payment of an annual premium. Health and hygiene programs at SKDRDP started as a *Jana Jagruthi* or public awareness program and included health awareness sessions at routine credit group meetings, home visits by a village health worker, the promotion of low cost sanitary latrines, and *Sampoorna Suraksha*, an insurance scheme with health cover. For the health insurance, an annual contribution of Rs. 190 (US $3) was collected from each member, providing protection for up to Rs. 5000 (US $83) in medical expenses per year.

These health programs were available to some, but not all, program areas of the two organizations. At the start of this study, half of the participating villages were identified for roll out of the health program – the intervention villages. For the purpose of this study, we selected matched comparison villages from the same block. The comparison villages were from the microfinance program areas with no health program. Village pairs were matched on four criteria: population size, SHG membership, location in the same block but not with a common boundary. The matching exercise was carried out primarily by the program managers from the participating organizations. To test the validity of the matching process, before the start of the health program we conducted a survey of the intervention and comparison villages to collect information regarding key socio-economic characteristics. These characteristics were compared to evaluate the effectiveness of the matching process.

Improving the health of mothers and children by improving the quality of sanitation and reducing financial burden due to illness were priority issues common to both organisations. Hence, five indicators were selected to assess the benefit of combining a health program with SHGs: diarrhoea among children, institutional delivery of babies, colostrum feeding to newborns, having a toilet at home, and money spent on treatment. These indicators were selected in consultation with the respective program managers of the two organizations.

The survey questions were defined in the following ways: diarrhoea in the youngest child less than two years old, and occurring in the two weeks preceding the survey; institutional delivery and feeding colostrum to newborn babies (for the youngest child less than two years old during the baseline survey and less than one year during the follow-up survey). The variable for money spent on treatment was an aggregate of four questions related to different types of medical expenditure and was asked for each family member.

Changes in episodes of diarrhoea in children is a sensitive indicator of health program effectiveness in the short-term.^12^, ^13^ Institutional delivery of babies is an important indicator in monitoring progress towards Millennium Development Goal five (to reduce the maternal mortality ratio by three quarters between 1990 and 2015).^14^ Not feeding colostrum to newborn babies, along with late initiation of breastfeeding and improper complementary feeding were found to be significant risk factors for underweight among children.^15^ The practice of open defecation poses a major challenge for health and safety in India, a fact acknowledged at the highest political level.^16^ With half of the population defecating in the open, there is a high risk of microbial contamination of water, which poses a major health risk. The indicator related to money spent on treatment was selected to capture reduction in out-of-pocket treatment expenditure across the study period. Indicators related to changes in mortality and morbidity such as neonatal mortality were considered for inclusion, but not included because of the limited sample size.

### Sampling and recruitment

Two rounds of the survey were conducted with 472 respondents: 219 from the intervention villages, and 253 from the comparison villages. Baseline data were collected before the roll out of the health program, and the follow-up survey was conducted with the same respondents after 12 months. The inclusion criteria at the time of the baseline survey were women of reproductive age having a child aged less than two years. An equal number of respondents were recruited from intervention and comparison villages. A list of SHG members in the intervention and comparison villages was made available by the participating organizations. Based on the list, we firstly identified eligible houses in each village. Based on the calculated sample size, we then selected households proportionate to size using systematic random sampling.

### Study tool and analysis plan

Face to face interviews were conducted with the respondents. A questionnaire was used to collect socio-economic information, general health status, and key indicators. Questions related to socio-economic variables were the same as those used in the District Level Household Survey – phase III,^17^ while the section on health expenditure was adapted from the National Sample Survey on Household Consumer Expenditure, which was conducted in all Indian states in 2009–10.^18^ The questionnaire was pilot-tested in villages that were not part of the study.

Responses from the three blocks were aggregated to perform the analysis. Disaggregated analysis by study sites was deemed inappropriate as sample sizes were too small if each of the two groups were analysed separately. The analysis followed two steps. A test of equality on the study variables was performed on the baseline survey data to assess the validity of the village matching process. This was done through chi square value of pooled estimates for intervention and comparison groups, and Wilcoxon equality of medians test where median value is reported.

A difference-in-difference analysis was performed to assess the additional impact of the health program, controlling for the baseline measures. The following explains our analysis:

Let intervention group (A)=1|0,time(T)=0|1.

Then Y={E[Y|A=1,T=0]−E[Y|A=0,T=0]}−{E[Y|A=1,T=1]−E[Y|A=0,T=1]}.

The regression equation then can be written as:

$$Y_{ij}=\alpha +\beta_{1}T_{ij}+\beta_{2}A_{ij}+\beta_{3}\left(T_{ij}\cdot A_{ij}\right)+X_{ij}+\varepsilon_{ij}$$

*Y* is the outcome of interest (five outcome variables), *A* takes the value one if respondent *i* from block *j* were from an intervention area (i.e. SHG with access to both microfinance and health program), *T* takes the value one if survey is conducted at the time of the one year follow-up period, *X* is a vector for control variables. The regression coefficient of interest is the interaction of intervention group and follow-up period, referred to as *β*_*3*_. This model assumes a common trend across the intervention and comparison groups, that is, in absence of the health program, the unobserved differences between the intervention and comparison groups would be same over time. As both the intervention and comparison group are matched at baseline, and are from the same block, assuming a common trend across the groups is reasonable.

Binary logistic regressions were performed on the binary outcome variables: institutional delivery, childhood diarrhoea, toilet at home and feeding colostrum to newborns. Adjusted odds ratios, with 95% confidence intervals, were reported as increased or decreased odds of the occurrence of an event. For the continuous outcome variable, money spent on treatment, a two-part model was applied: first, respondents who had no expenditure on treatment in the previous month were identified, and then a linear regression was performed among those respondents who had spent money on treatment in the previous month. Per capita monthly expenditure on treatment was calculated by dividing the total expenditure on treatment by the number of family members in the household. Monetary values are reported in US$ with one US$ corresponding to 60 Indian Rupees.

All regression equations were controlled for respondents education, types of house (permanent, semi-permanent, or temporary structure), and monthly household expenditure. The three blocks included in the study have different socio-economic status which may have confounded the result. Hence we included the blocks as a categorical variable to control for the block effects.

### Focus group discussions and key informant interviews

A qualitative study was conducted after the follow-up survey in the intervention villages to understand the contextual factors and challenges associated with the health program. In total, 17 key informant interviews with program managers and village health workers (VHWs), and 17 focus group discussions (FGDs) involving 153 community members were conducted in order to achieve data saturation. Both the FGD and key informant guides were designed to seek information on three themes: community context, group structure and functioning, and contribution of the health program. All interviews were conducted in the local language.

## Results

### Characteristics of the participants

A description of the socio-economic characteristics of the sample at baseline is presented in Table 1. The median age of respondents in the intervention group was 28 years (range: 23–33) and in the comparison group was 29 years (range: 24–34). A quarter of respondents (27.4%) lived in a permanent or *pucca* house. Respondents from Udupi had better housing, compared to Gadag and Dahegam. The majority of respondents (73% in intervention group, and 77% in comparison group) had access to tap-water, either at their own house or from a near-by public source. Again, respondents from Udupi and Dahegam had better access to tap-water, compared to respondents from Gadag. A quarter of the respondents did not have any formal education. Average monthly household expenditure was US$ 73 for both groups.

The intervention and comparison groups were not significantly different on key socio-economic variables: type of housing, access to piped water, and average monthly household expenditure. This supports the validity of the village matching process.

Among the 17 key informants interviewed for the qualitative study, three were program managers (one from each program block) while the rest were village health workers. Additionally, 17 FGDs were conducted with a total of 153 participants. The mean age of the participants was 28 years (range: 25–30) (Table 2).

### Program impact

Compared to the comparison group, SHG members with a health program had higher odds of delivering their babies in an institution, feeding colostrum to newborns, and having a toilet at home after one year of program implementation. However, the SHG plus health program group showed no significant improvement in the incidence of diarrhoea among children and no effect on money spent on treatment. The results were adjusted for pre-program measures, and socio-economic characteristics of the household. The following section discusses the results for each of the five selected indicators.

Before implementation of the health program, 76.4% (149/195) of women in the intervention villages, and 80.0% (184/230) of women in the comparison villages reported delivering their most recent baby in an institution. At follow-up, 70 women from the intervention villages, and 93 women from the comparison villages had a delivery experience. The proportion of respondents reporting delivery in an institution during the 12 months preceding the follow-up survey rose in both groups, but significantly more so in the intervention group (from 76.4% to 95.7%) than in the comparison group (from 80.0% to 86.0%). The difference between groups after adjustment for baseline values was significant (OR: 5.08, 95% CI 1.21–21.35) (Table 3) suggesting that the combination of a health program with a SHG was associated with an increase in institutional delivery of babies. The study hypothesis that the health program would result in an increase in institutional delivery is supported by this result.

Before implementation of the health program, 56.3% (103/183) of respondents in the intervention villages, and 59.3% (118/199) of respondents in the comparison villages reported feeding colostrum to their newborns. During the follow-up survey feeding colostrum to newborns was reported for babies born during the 12 months follow-up period: 70 respondents from the intervention villages, and 93 respondents from the comparison villages. There was a larger increase in the proportion of newborns fed colostrum in the intervention group compared to the comparison group; the percentage went up from 56.3% to 77.5% in the intervention group and from 59.3% to 62.0% in the comparison group. There was a statistically significant difference between the intervention and comparison groups at the follow-up period, after adjustment for baseline characteristics (OR: 2.83, 95% CI 1.02–5.57) (Table 3). The study hypothesis that the health program would result in an increase in feeding colostrum to newborns is supported by this result.

Before the start of the health program, 62.6% (137/219) of respondents in the intervention villages and 51.8% (131/253) of respondents in the comparison villages reported having a toilet at home. This is more than the estimate of 46.9% toilet ownership in India as per the 2011 census.^19^ The proportion increased slightly from 62.6% to 65.8% in the intervention group and was essentially unchanged at 51.8%–50.6% in the comparison group. The difference between groups after adjustment for baseline values was in the expected direction, although not statistically significant (OR: 1.53, 95% CI 0.76–3.09) (Table 3). While survey results highlight the effect of the health program on toilet ownership, qualitative interviews highlighted some of the challenges faced by women due to lack of access to toilet:

*Having no toilet is an insult to women. We are forced to defecate in the open field. If males are walking on the road, we have to stand. This is shaming. It was not easy. After attending the sessions on cleanliness and personal hygiene, I decided that for the sake of my two adolescent daughters I needed a small toilet. I had to convince my husband and my in-laws of the need to have a toilet of our own. (FGD, Gadag)*.

Before implementation of the health program, 26.0% (57/219) of respondents in the intervention villages, and 25.3% (64/253) of respondents in the comparison villages had a child who suffered from diarrhoea in the preceding two weeks. This fell to 11.0% (24/219) and 12.6% (32/253) at the time of the follow-up survey. The proportion of children suffering from diarrhoea went down in both the intervention and comparison villages. While respondents from villages in the intervention group had 14% lower odds of having a child suffering from diarrhoea in the study reference period compared to those in the comparison villages the difference was not statistically significant (OR: 0.86, 95% CI 0.42–1.76) (Table 3). Nevertheless, during FGDs, women attributed the reduction in diarrhoea episodes to the awareness generated through the health program.

*This year we had fewer cases of diarrhoea among children, compared to the same period last year. I would count this as a success. We are more aware about how to keep children clean, how to assist growing children, what food should be given, feeding boiled water to small children, etc. She [NGO health worker] advises us to give salt-sugar solution and ORS if any child suffers from diarrhoea. (FGD, Udupi)*.

About 40% of the respondents surveyed at both time points reported no expenditure on treatment for health problems in the previous month. Among respondents who had spent money on treatment, the per capita spending at baseline was higher in the comparison group than in the intervention group (US$ 6.64 in the intervention group compared to US$8.80 in the comparison group). This declined to US$ 3.93 in the intervention group, and to US$ 4.19 in the comparison group. The adjusted estimates suggest that the study hypothesis that the health program would result in a reduction in money spent on treatment is not supported by this result (Table 3).

### Contextual factors and challenges

The qualitative study among the intervention villages focussed on understanding the community context, experience in implementing the health program, and challenges in program implementation. The following section discusses the findings from the qualitative study.

### Rationale for the health program

Program managers reported that primary health care services in their villages were limited; government health facilities were either not present or, if present, were under-resourced. Moreover, poor health awareness meant that people often resorted to unqualified providers for care, or did not adopt appropriate approaches to prevent diseases. For example, people defecated in the open, women had poor sanitary practices, and traditional beliefs about child care practices were common.

Some program managers said that the primary business of a microfinance institution is to lend money to SHG members. This money is meant to develop businesses that can be used to generate income. While investing the borrowed money for business expansion was an important option for self-sufficiency of members, such a mechanism sometimes met with limited success due to illness and lack of health awareness among the SHG members. Loans taken for generating income were being used to meet the cost of treatment for health problems if someone in the house fell sick. Respondents reported that some SHG members were occasionally unable to repay their loans due to illness.

*They spend their meagre resources on food that barely meets their nutritional requirements. Malnutrition and sickness force them to contain health spending, and they are unable to even seek treatment thereby reducing their family income. This creates a vicious cycle. It is impossible to escape the clutches of poverty. Poor health is one of the biggest contributors to poverty; members needed awareness of good health, appropriate and affordable healthcare options. (KII, Program Manager, Gadag)*.

The program was designed around a cadre of VHWs, nominated by the SHGs. The VHWs worked to raise awareness of reproductive and child health, immunization and childcare, hygiene and sanitation; to refer people with danger signs of pregnancy and child health complications; and to promote a health insurance product to cover health-related consultations and treatments. Many VHWs said that the contents of the training sessions challenged some of their own misguided health beliefs, and this learning was subsequently shared during the SHG meetings. Some respondents reported that non-members were also encouraged to participate in the health education sessions:

*Through training we gained knowledge about family planning, diarrhoea among children, immunization, breast feeding, diet of the mother, and how to maintain hygiene within the community. My own beliefs about child care practices have changed as a result of the training. (KII, Village Health Worker, Udupi)*.

*After our regular [SHG] meeting, we organized a discussion on one topic at a time. Some meetings focused on diarrhoea and cleanliness, some focused on health of girls in our community, while others focused on sanitation. We reinforced the health messages during home visits. (KII, Village Health Worker, Gadag)*.

### Trust and social capital

A strong and common theme emerging from the interviews and discussions was trust and solidarity between group members and with their respective organizations. Respondents attributed their trust and confidence with the participating organizations to their origins: one of the organizations is associated with a famous and respected temple trust in Udupi (SKDRDP), while another emerged as a trade union for self employed women (SEWA). Both organizations had been involved with microcredit activities in the study areas for over a decade. Another common theme emerging from the discussions was members' belief that their groups were based on the principles of equality, trust, discipline, respect and helping each other. Members believed that the group leader played a key role in setting up and maintaining the group values and norms.

*Our group is formed on the principle of cooperation, trust, and respect…By joining the SHG I am happy. Earlier if I asked for Rs. 10 from my husband, I used to get a scolding. After opening accounts with the group, I am also getting interest on my savings. I also got Rs. 50,000 [US $833] as a loan for my daughter’s marriage. (FGD, Udupi)*.

Over the course of discussions, participants described the ways in which the organizations influenced their daily lives. SHG meetings acted as a platform for discussing issues that commonly concerned the communities, such as education of children, access to safe drinking water, sanitation, and illness. Respondents also narrated incidents where the organizations provided material assistance to help them solve local issues such as support for setting up a local water treatment plant, constructing toilets at home, and setting up milk dairy cooperatives that were then maintained by SHG members. However, there were larger issues such as improvements in road and drainage infrastructure, issues related with employment and farming that could not be addressed at the SHG level. These issues were raised with the village panchayat (local government in the Indian subcontinent) by the SHG leaders and organization representatives.

*We used to face difficulty in treating water for drinking. The water obtained from the well is not suitable for drinking. SKDRDP supported us in setting up a water treatment plant in our village. Our group now runs the plant. One of us is trained in maintaining the plant. We also sell the water at low-cost in our village. (FGD, Udupi)*.

### Challenges in health program implementation

The interviews and FGDs highlighted several challenges encountered by VHWs while delivering the health program. It was difficult to change behaviours that are deeply entrenched in the community. The program design and delivery also presented a challenge in some places.

### Challenges related to the community context

In-depth interviews with key informants identified some of the challenges that negatively affected efforts to achieve behaviour change. These included: traditional beliefs about health and illness; relying on unqualified traditional healers as the first point of care resulting in delayed care seeking from the formal health service; and money wasted when seeking care from unqualified health practitioners. On respondent said:

*They usually take medicine from a small hut/shop. If they cannot see any change then they go to a qualified doctor. We do constantly remind them to go to a qualified doctor. However, it is usually the head of the family who takes the decision. Also there are superstitions and religious beliefs that stop them from taking care. (KII, Program Manager, Dahegam)*.

The primary recipients of microcredit are women, and hence the health programs were developed with women as the primary target group. While participants believed the women-centric approach has promoted their participation in household decision making and control over resources, they also highlighted instances where control over resources was the cause of tension and intimate partner violence within the family, particularly when males felt that their dominance was being challenged. SHG members called for programs that also involved males in the behaviour change process, particularly related to alcohol and substance abuse, and risky sexual practices.

### Programmatic challenges

VHWs highlighted several programmatic challenges in delivering the health program. Some challenges related to the way they were compensated, while others related to procedural issues. In some instances, the VHWs complained that the honorarium was not sufficient to compensate for the responsibilities associated with the program, while for others motivating the community to change their long held beliefs and behaviours was difficult. One respondent said:

*We have a long way to go. Our members have faith in us. However, others doubt our intention. There are ignorance and wide-spread superstitions. Initially we faced difficulties in convincing people to change their behaviours. (KII, Village Health Worker, Dahegam)*.

There were also procedural delays in processing SHG members' health insurance claims. This contributed to poor perception of the health insurance product. There were instances of delays in receiving reimbursement after discharge from hospital, as administrative staff at the hospital did not always cooperate in providing the information or documentation required to process the claim. Sometimes the beneficiaries did not carry the required documentation (for example an insurance card) with them to the hospital. This led to delays in authorization of the payment procedure at the hospital. As one respondent reported:

*There were too many delays in processing the health insurance claims. We come from faraway places. When the delay occurs we have to miss our bus to go back home. (FGD, Gadag)*.

However, the program managers also mentioned that, overtime, they had succeeded in overcoming most of the initial hurdles through a system of routine feedback to the VHWs, dialogue with concerned stakeholders, and training. These activities had resulted in members feeling encouraged to increase the frequency and quality of participation in group activities.

Program managers and VHWs emphasized the need for more interaction with local health officials to upgrade their own knowledge related to health issues affecting their communities. Some of the critical issues identified for skill building were related to adolescent health issues, sexually transmitted infections, and government programs and schemes operational in their villages.

On being asked about future program efforts, program managers stressed the need to promote cleanliness in their villages, work with local authorities to build and maintain drainage systems, and strengthen programs that aimed to stop the practice of open defecation. Participation of males in the programs was listed as another priority area. Additionally, the need to attend to broader development issues such as creating opportunities for employment, and training in vocational skills were highlighted during the course of interviews with the program managers.

## Discussion

The findings from this study indicate that compared to a matched comparison group, an intervention combining a health program with microfinance-based SHG activities positively influences some, but not all, health behaviours and outcomes over a one-year follow-up period. Adjusting for baseline measures, and controlling for respondents' education, type of house, monthly household expenditure, and geographical location, being a member of a village with an SHG health program was associated with a higher odds of delivering their most recent baby in an institution, feeding colostrum to their newborn babies, and having a toilet at home, compared to a matched comparison group.

The effects observed in our study are consistent with existing evidence. However, most of the existing evidence found changes in a controlled research setting, while our study was in a real-life setting associated with real-life challenges to implementation.

The SHG structure emphasizes social cohesion and promotes collective action related to members shared needs. While the health programs in the study villages were new initiatives, both organizations (SEWA and SKDRDP) had implemented microcredit activities in those villages for more than a decade. Long duration of association with the community, SHG structure, and reputation of the organization seems to have played a crucial role in promoting trust for their organization among members. Personal bonds and trust of SEWA members has been documented in several ethnographic studies.^20^, ^21^ Trust and social capital as a result of members' participation in SHG activities echoes findings widely documented in the literature.^22^, ^23^, ^24^

While change occurred in the expected direction, there was no evidence of a statistically significant reduction in diarrhoea among children in the intervention community, and the study hypothesis that the health program would result in a reduction in money spent on treatment was not supported by the results. Possible reasons for this mixed effect could relate to the way the health program was delivered. In the absence of a process evaluation of the health program we have limited information on the program's content, quality, and frequency of delivery across the different sites. An explanation for the lack of success in relation to some indicators could be the fact that the VHWs were inexperienced – being the first year of program implementation, they were new in their role so had not had time to consolidate their knowledge and gain confidence. Time and mentoring may be necessary for VHWs to learn to function effectively in this role and to gain respect for their knowledge. More changes may have been evident with a longer follow-up period. Additionally, our qualitative findings highlighted initial challenges in motivating the community, especially issues with honorarium payments for VHWs, delays in processing health insurance claims, and delays in seeking care from qualified healthcare providers due to traditional beliefs about health and illness. While several of these hurdles were ultimately addressed, a longer period of time would be required to implement the health program in its entirety than was possible in this study. This mixed program effect is, however, not uncommon among completed research on this subject.^25^, ^26^, ^27^, ^28^

### Conclusion

Our study found evidence that combining a health program with microfinance-based SHG activities is associated with a significant increase in women delivering their babies in an institution, feeding colostrum to their newborn, and a non-significant increase in having a toilet at home. However, the program did not produce a significant change in the outcome indicator related to diarrhoea among children, and had no effect in reducing money spent on treatment.

With broad population coverage, microfinance-based SHGs provide an avenue for increasing universal health coverage and particularly for addressing the health needs of poor women. Our results indicate that further research on this theme is required. There are additional reasons, from a social perspective, for investigating the possible positive impact of these programs. These include the impact of broad population coverage provided by SHGs and the social capital produced by their activities. A key area of future research would be an assessment of cost of adding a health program to SHGs more widely, and an analysis of cost-effectiveness of such an integrated approach. Public health planners stand to benefit from the membership-based structures and social capital that already exist through microfinance-based SHGs. However, such programs should not be viewed as a panacea for government failures. Rather, the SHG-based programs can be seen as complementary to public provisioning of health services, and as a means for increasing awareness about entitlement for public services in the community.

## Author statements

### Acknowledgement

Dr. James Hargreaves from the London School of Hygiene and Tropical Medicine provided useful guidance in the study design. Thanks are due to the NGO program managers, village health workers and study participants for sharing their experience.

### Ethical approval

The interview process was explained to respondents in plain and relevant vernacular language. Before initiating the survey, interviews or FGDs, written informed consent was obtained from eligible respondents who were literate. Verbal informed consent was obtained from women who were illiterate, after the purpose and proceedings of the study were explained to them. The research protocol for this study received approval from both the Nossal Institute for Global Health Human Ethics Advisory Group at the University of Melbourne, Australia (Ethics Id: 1239067.1), and the Institutional Ethics Committee of the Public Health Foundation of India, New Delhi, India (TRC-IEC-124/12).

### Funding

The research was supported by a Welcome Trust Capacity Strengthening Strategic Award to the Public Health Foundation of India and a consortium of UK Universities, and a Research Higher Degree grant from the Nossal Institute for Global Health at The University of Melbourne, Australia. The funders of the study had no role in study design, data collection, data analysis, data interpretation, or writing of the report. The corresponding author had full access to all the data in the study and had final responsibility for the decision to submit for publication.

### Competing interests

None.

## Figures and Tables

**Table 1:** Characteristics of respondents at baseline.

	Intervention group	Comparison group	*P*-value
**Villages**			
Number of villages enrolled	17	17	
Type of house			0.12
Permanent *(Pucca)* house	68 (27.4%)	58 (26.7%)	
Semi permanent (Semi-*pucca)* house	125 (50.4%)	116 (53.0%)	
Temporary *(Kutcha)* house	55 (22.2%)	43 (20.0%)	
Proportion of household with access to tap water	160 (73.0%)	177 (70.0%)	0.16
**Individuals**			
Number of respondents interviewed at baseline	219	253	
Age of respondent (median in years, IQR)	28 (23–33)	29 (24–34)	0.35
Education			0.10
No formal education	59 (24.0%)	57 (26.6%)	
Education: 1–8 grade	127 (51.6%)	101 (47.0%)	
Education: 9–12 grade	49 (19.9%)	45 (21.0%)	
Education: more than 12 grade	11 (4.5%)	11 (5.1%)	
Monthly household expenditure (mean in US$)	73	73	0.92

**Table 2:** Characteristics of key informants and focus group discussion participants.

**Key informant interviews**	**17**
Gender of participants	
Male	1
Female	16
Role of participants	
Program manager	3
Village health worker	14
**Focus group discussion**	**17**
Gender of participants	
Female	153
Mean age (years)	28

**Table 3:** Difference-in-difference effect of the health program on measured indicators.

	Baseline		Follow-up		Unadjusted odds ratio	Adjusted odds ratio
Intervention group	Comparison group	Intervention group	Comparison group
Institutional delivery	149/195 (76.4%)	184/230 (80.0%)	67/70 (95.7%)	80/93 (86.0%)	4.48 (1.13–17.75)	5.08 (1.21–21.35)
Feeding colostrum to newborns	103/183 (56.3%)	118/199 (59.3%)	55/70 (77.5%)	57/93 (62.0%)	2.39 (1.06–5.36)	2.38 (1.02–5.57)
Toilet at home	137/219 (62.6%)	131/253 (51.8%)	144/219 (65.8%)	128/253 (50.6%)	1.20 (0.71–2.03)	1.53 (0.76–3.09)
Diarrhoea among children	57/219 (26.0%)	64/253 (25.3%)	24/219 (11.0%)	32/253 (12.6%)	0.82 (0.41–1.65)	0.86 (0.42–1.76)
Respondents that reported nil expenditure on treatment in previous month	83/219 (37.9%)	107/253 (42.3%)	79/219 (36.1%)	107/253 (42.3%)	–	–
Per capita mean monthly spending in USD on treatment (SD), among respondents who had spent money on treatment	6.64 (4.43)	8.80 (6.30)	3.93 (10.02)	4.19 (10.04)	1.0	1.85

Results are from binary logistic regression (odds ratio with 95% confidence interval), and linear regression coefficient. Coefficient of interest is the interaction of study arm and study round, which is adjusted for baseline measures, respondent's education, type of house, monthly household expenditure, and blocks. Some variables had missing data. In case of the variables: institutional delivery and feeding colostrum to newborn, figures in the follow-up period refers to respondents who had a delivery experience over the 12 months follow-up period.
